# Importance of accurate and accessible recording of healthcare contacts in mental health

**DOI:** 10.1186/s12916-023-03044-w

**Published:** 2023-09-12

**Authors:** Ruth H. Jack, Carol A. C. Coupland, Rebecca M. Joseph, Chris Hollis, Richard Morriss, Roger David Knaggs, Andrea Cipriani, Samuele Cortese, Julia Hippisley-Cox

**Affiliations:** 1 Centre for Academic Primary Care, School of Medicine, University of Nottingham, Nottingham, UK; 2 National Institute of Health and Care Research MindTech MedTech Co-Operative, The Institute of Mental Health, University of Nottingham, Nottingham, UK; 3 National Institute of Health and Care Research Nottingham Biomedical Research Centre, Nottingham University Hospitals NHS Trust, Nottingham, UK; 4 Mental Health and Clinical Neurosciences, University of Nottingham, Nottingham, UK; 5 School of Pharmacy, University of Nottingham, Nottingham, UK; 6 Department of Psychiatry, University of Oxford, Oxford, UK; 7 Oxford Health NHS Foundation Trust, Warneford Hospital, Oxford, UK; 8 Oxford Precision Psychiatry Lab, NIHR Oxford Health Biomedical Research Centre, Oxford, UK; 9 Center for Innovation in Mental Health, School of Psychology, Faculty of Environmental and Life Sciences, Clinical and Experimental Sciences (CNS and Psychiatry), Faculty of Medicine, University of Southampton, Southampton, UK; 10 Solent NHS Trust, Southampton, UK; 11 Department of Child and Adolescent Psychiatry, Hassenfeld Children’s Hospital at NYU Langone, New York, NY USA; 12 Nuffield Department of Primary Care Health Sciences, University of Oxford, Oxford, UK

**Keywords:** Mental health, Electronic health records, Data availability, Primary care, Secondary care, Children and young people

Thank you for drawing our attention to the correspondence article by Taxiarchi et al. [1]. Our original study [2] used linked primary care electronic health records (QResearch) and secondary care data (Hospital Episode Statistics) to assess whether there were records of children and young people (CYP) visiting NHS-funded paediatric or psychiatric specialists in secondary care within the 12 months before or up to 6 months after their first primary care antidepressant prescription.

Taxiarchi et al.’s [1] work looks at a group of CYP in the Clinical Practice Research Datalink (CPRD) in a similar period who had a coded record in their primary care data of being “Seen in child and adolescent psychiatry clinic” or “Seen by child and adolescent psychiatrist” and identifies whether there was a relevant inpatient or outpatient hospital episode recorded in the 12 months before this. This estimates that 27.5% (or at most, 56.0% in a sensitivity analysis looking at 24 months before the primary care record) of those with a CPRD record of having seen a child and adolescent psychiatrist had a corresponding HES record. We highlighted the limitation in our paper that it was “possible that we did not capture all interactions with specialists” [2], and Taxiarchi et al.’s [1] work may point to the extent of contact with the private sector, which is not included in HES data.

These studies are looking at different outcomes for different populations in different datasets, but both highlight the importance of accurate and accessible recording of healthcare contacts in order to describe and quantify healthcare utilisation. In response to the commentary article published at the time [3], we highlighted the information gap that exists when trying to assess issues around mental health care, in particular for children and adolescents [4]. When the Mental Health Services Dataset (MHSDS) was available as linked data to CPRD, it did not contain information from Child and Adolescent Mental Health Services (CAMHS) [5].

## Data Availability

Not applicable.
